# Core Ferroptosis-Related Biomarkers and miRNA Regulatory Networks in Alzheimer’s Disease

**DOI:** 10.3390/genes17020224

**Published:** 2026-02-11

**Authors:** Wenjia Liu, Xin Rao, Liyang Yu

**Affiliations:** 1 School of Electronic Information Engineering, Suzhou Polytechnic University, Suzhou 215104, China; 2 School of Electronics and Information, Hangzhou Dianzi University, Hangzhou 310018, China; raoxin1992@hdu.edu.cn

**Keywords:** Alzheimer’s disease, ferroptosis, differentially expressed genes, miRNA, biomarker, regulatory network

## Abstract

**Background**: The exact pathogenesis of Alzheimer’s disease (AD), a neurodegenerative disorder, remains unclear. Ferroptosis is a form of cell death characterized by intracellular iron accumulation, and has emerged as a potential contributor to the pathological cascade of AD. Therefore, this study aims to identify core genes that may function as reliable biomarkers for AD through an in-depth analysis of the genetic relationship between ferroptosis-related genes and AD. **Methods**: This study first obtained the gene expression profiles (GSE140831, GSE63060 and GSE63061 expression profiles). The GSE140831 dataset served as the discovery cohort, and the GSE63060 and GSE63061 datasets were used as independent validation cohorts. R language 4.4.1 was used for standardizing and identifying differentially expressed genes (DEGs) in AD patients in all datasets. Secondly, the ferroptosis-related genes were obtained. By integrating the ferroptosis-related genes, ferroptosis-related DEGs (FRDEGs) were detected. Then, the FRDEGs were verified and evaluated, and the biological functions of the core genes were analyzed. Finally, miRNAs interacting with these core FRDEGs were explored. **Results**: The study identified nine FRDEGs (ACVR1B, BRPF1, G6PD, KLHDC3, LAMP2, MTCH1, P4HB, PTPN6, RBMS1), which are potentially related and may serve as biomarkers for AD. All nine genes demonstrated statistically significant differential expression (up-regulation) in both independent validation cohorts and in the combined analysis (*p* < 0.05). Although the area under the curve (AUC) values of these nine genes ranged from 0.61 to 0.71, indicating moderate discriminatory power, these findings suggest that they may be involved in pathways related to AD and are worthy of further investigation as potential auxiliary biomarkers. Finally, a network of hub FRDEGs-miRNAs interaction was constructed. There were 11 miRNAs that may regulate these hub FRDEGs simultaneously. **Conclusions**: This study showed the significant association of the identified FRDEGs with AD. Also, a core ferroptosis-related biomarker network for miRNAs regulation of AD was constructed. The specific regulatory mechanism is worthy of further investigation.

## 1. Introduction

As a predominant neurodegenerative disorder, Alzheimer’s disease (AD) has seen a growing global prevalence due to extended life expectancy and aging populations, imposing substantial societal and familial challenges. The exact pathogenic mechanisms of AD remain incompletely understood despite decades of investigation [1]. Extracellular amyloid plaques formed by beta-amyloid (Aβ) and intracellular neurofibrillary tangles (NFTs) composed of hyperphosphorylated tau proteins are the key pathogenic characteristics of AD. For decades, considerable research has been conducted regarding the Aβ and tau proteins, but progress in the treatment of AD has been limited [2]. Current studies have shown a connection between the process of ferroptosis and various neurological illnesses, including participation in AD pathology [3,4]. Ferroptosis is an iron-dependent cell death pathway [5,6] that can lead to oxidative stress within the cell, damage proteins, nucleic acids, etc., and ultimately result in cell death [7].

Ferroptosis markers, such as lipid peroxidation and disrupted iron metabolism, have been consistently observed in the brains of AD patients [8,9]. Glutathione peroxidase 4 (GPX4) acts as a regulator that can induce ferroptosis. In an animal study, mice with cortical and hippocampal neuron-specific GPX4 knockouts were found to show significant cognitive impairment [10]. In addition, the degenerative neurons may be undergoing ferroptosis. The study found that feeding mice with the ferroptosis inhibitor (liproxstatin-1) could reduce the severity of neurodegenerative diseases [10]. Another study showed that the excessive expression of tau protein induced the death of iron-addicted neurons. This neuronal loss can be rescued by down-regulating the transferrin receptor, reducing p-P38 levels, and up-regulating GPX4 expression [11]. According to these studies, it has become clear that ferroptosis has significantly affected the neurons that are responsible for cognitive functions. It is further suggested that ferroptosis represents a distinct cell death pathway in AD pathogenesis, contributing significantly to neurodegeneration during disease progression [12]. However, further investigation is necessary to establish this hypothesis with certainty.

Current investigations into ferroptosis in AD have focused on evaluating the therapeutic potential of ferroptosis inhibitors, particularly their ability to modify disease progression. However, there still is no consensus on the genetic mechanism among them [13,14,15]. Therefore, this study aims to identify critical genes that may function as reliable biomarkers for AD by conducting an in-depth analysis into the relationship between ferroptosis-related genes and AD at the genetic level.

## 2. Materials and Methods

**Figure 1:**
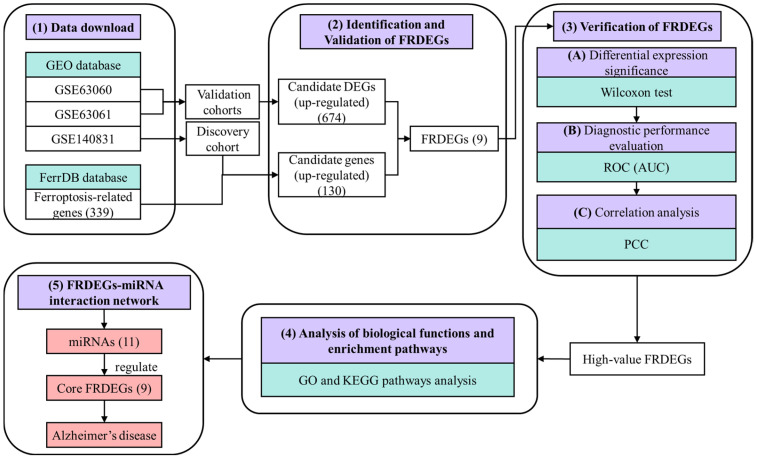
The overall workflow of this study. GEO: Gene Expression Omnibus; PCC: Pearson correlation coefficient; ROC: receiver operating characteristic; AUC: area under the curve; GO: Gene Ontology; KEGG: Kyoto Encyclopedia of Genes and Genomes. Purple indicates the analysis purposes; green indicates the data sources and analysis tool; pink indicates the final significant result, namely miRNAs regulate nine core FRDEGs associated with AD.

The overall workflow of this study is shown in Figure 1. In this work, gene expression profiles (GSE140831, GSE63060 and GSE63061) and ferroptosis-related genes were first downloaded. The GSE140831 dataset served as the discovery cohort, and the GSE63060 and GSE63061 datasets were used as independent validation cohorts. Then, differentially expressed genes (DEGs) were identified, and key ferroptosis-related differentially expressed genes (FRDEGs) were identified and verified through the integration of gene expression profiles. Subsequently, the key FRDEGs were further validated, including verification of significant differential expression levels, assessment of potential significance, and correlation analysis. And the biological function and enrichment pathway of high-value FRDEGs were analyzed. Finally, the hub FRDEGs-miRNA interaction network was constructed, and the miRNA-regulating core FRDEGs were predicted, suggesting ferroptosis as a viable AD treatment target.

### 2.1. Data Acquisition and Preprocessing

The GSE140831, GSE63060 and GSE63061 datasets were downloaded from the GEO database (http://www.ncbi.nlm.nih.gov/geo/ (accessed on 5 February 2026)) [16,17]. These datasets are derived from blood samples of AD patients and control subjects. The GSE140831 dataset included 338 AD patients and 530 control subjects, the GSE63060 dataset included 145 AD patients and 104 control subjects, and GSE63061 dataset included 140 AD patients and 135 control subjects. The statistics are presented in Table 1. Ferroptosis-related genes were derived from the ferroptosis-related database (FerrDB) (http://www.zhounan.org/ferrdb/current/ (accessed on 5 February 2026)) [18], including ferroptosis_driver, ferroptosis_suppressor and ferroptosis_marker (gene with protein product).

### 2.2. Screening Strategy for FRDEGs

To ensure robust and replicable findings, this study adopted a two-phase analytical strategy comprising a discovery phase and an independent validation phase. Discovery phase: The GSE140831 dataset served as the discovery cohort. The DEGs between AD patients and control subjects were standardized and analyzed using the “limma” package in R language 4.4.1. Gene expression values were normalized within this dataset. Genes with an adjusted *p*-value (false discovery rate, FDR) < 0.05 and |logFC| > 0 were considered significant DEGs [19]. These DEGs were then intersected with the curated list of ferroptosis-related genes obtained from the FerrDB database to identify candidate FRDEGs for further validation. Validation phase: The GSE63060 and GSE63061 datasets were used as independent validation cohorts to assess the reproducibility of the candidate FRDEGs identified in the discovery phase.

### 2.3. Verification of the Candidate FRDEGs in Independent Cohorts

The candidate FRDEGs identified from the discovery cohort (GSE140831) were further validated in the two independent validation cohorts (GSE63060 and GSE63061). The validation process included the following analyses, which were performed separately within each validation dataset. (A) Differential expression significance: A Wilcoxon test (using the “ggpubr” package in R language) was employed to confirm the significant differential expression of each candidate FRDEG between AD patients and control subjects (*p*-value < 0.05) [20]. Prior to this analysis, z-score normalization was applied to the gene expression values within each individual dataset to minimize potential batch effects. (B) Diagnostic performance evaluation: The potential diagnostic utility of each candidate FRDEG was assessed using ROC analysis (using the “pROC” package in R language). The AUC and its 95% confidence interval (CI) were calculated for each gene within each validation cohort (GSE63060 and GSE63061) separately. An AUC value > 0.5 indicates predictive ability beyond chance [21]. (C) Correlation analysis: To explore the potential interconnectedness among the validated FRDEGs, PCC analysis was conducted for each validation cohort using an online platform (https://www.bioinformatics.com.cn (accessed on 5 February 2026)).

### 2.4. Biological Functional Analysis of FRDEGs

GO analysis on FRDEGs using R language (“clusterProfiler” package) (*p*-value < 0.05) was applied to further understand the potential functions, which included biological process, molecular function, and cellular component [22]. Additionally, KEGG pathway analysis was conducted using R language (“pathview” package) (*p*-value < 0.05) to identify the signaling pathways for gene enrichment [23].

### 2.5. Visual Network Construction of FRDEGs-miRNA

The visual network of FRDEGs-miRNA interactions was constructed through the TarBasev9.0 database on the Networkanalyst website [24]. The parameters used included the specific organism (*H. sapiens*, human) and set ID type (Official Gene Symbol).

## 3. Results

### 3.1. Identification and Validation of FRDEGs

**Figure 2:**
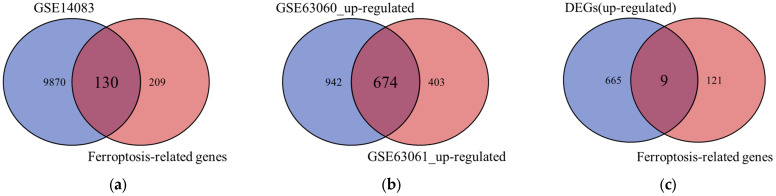
The identification of DEGs and FRDEGs. (**a**) Identification of candidate ferroptosis-related genes from the discovery cohort (GSE140831). (**b**) Consistently up-regulated DEGs in the independent validation cohorts (GSE63060 and GSE63061). (**c**) Core FRDEGs validated across both discovery and validation phases.

Differential gene analysis was first performed between AD patients and control subjects in each of the three cohorts. The differential analysis results showed that the GSE140831, GSE63060 and GSE63061 datasets revealed 10,000 DEGs (1768 up-regulated and 8232 down-regulated), 3053 DEGs (1616 up-regulated and 1437 down-regulated), and 1987 DEGs (1077 up-regulated and 910 down-regulated), respectively. To identify candidate ferroptosis-related markers, the 10,000 DEGs from the discovery cohort (GSE140831) were intersected with a curated list of 339 human ferroptosis-related genes. This initial screening yielded 130 candidate genes, all of which were up-regulated, as shown in Figure 2a. Subsequently, to validate and refine these candidates, this work identified the common up-regulated DEGs shared between the two independent validation cohorts. The intersection of up-regulated genes from GSE63060 and GSE63061 yielded 674 consistently up-regulated genes, as shown in Figure 2b. Finally, to pinpoint the most robust and replicable ferroptosis-related signatures, the 130 candidate genes from the discovery phase were cross-referenced with the 674 consistently up-regulated genes from the validation phase. This integrative analysis identified nine core genes that were significant in both the discovery and validation stages, as shown in Figure 2c. These nine genes (ACVR1B, BRPF1, G6PD, KLHDC3, LAMP2, MTCH1, P4HB, PTPN6, and RBMS1) were thereby defined as the key FRDEGs for AD in this study. All nine FRDEGs were up-regulated in AD compared to controls; the specific information is statistically presented in Table 2.

### 3.2. Validation of FRDEG Expression in Independent Cohorts

**Figure 3:**
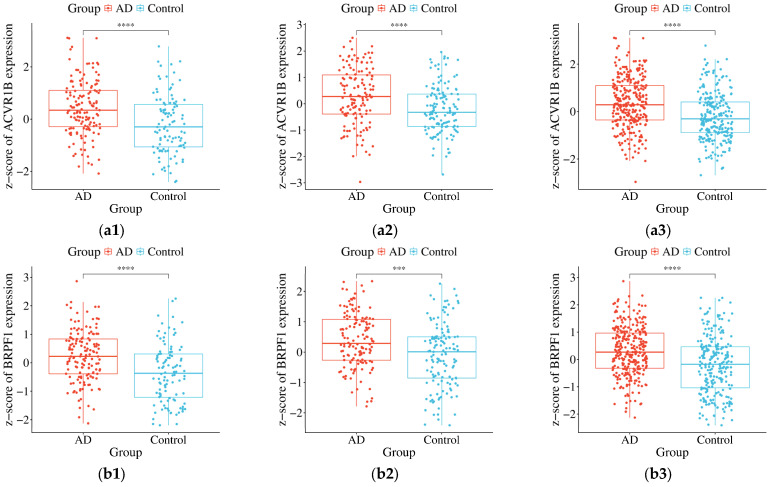

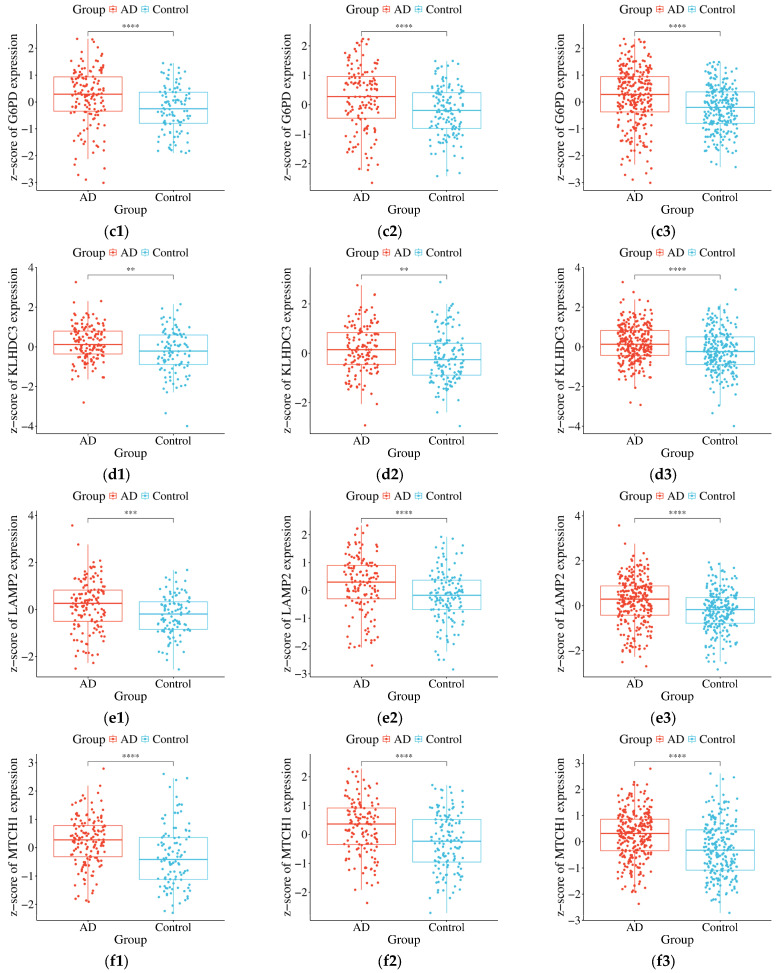

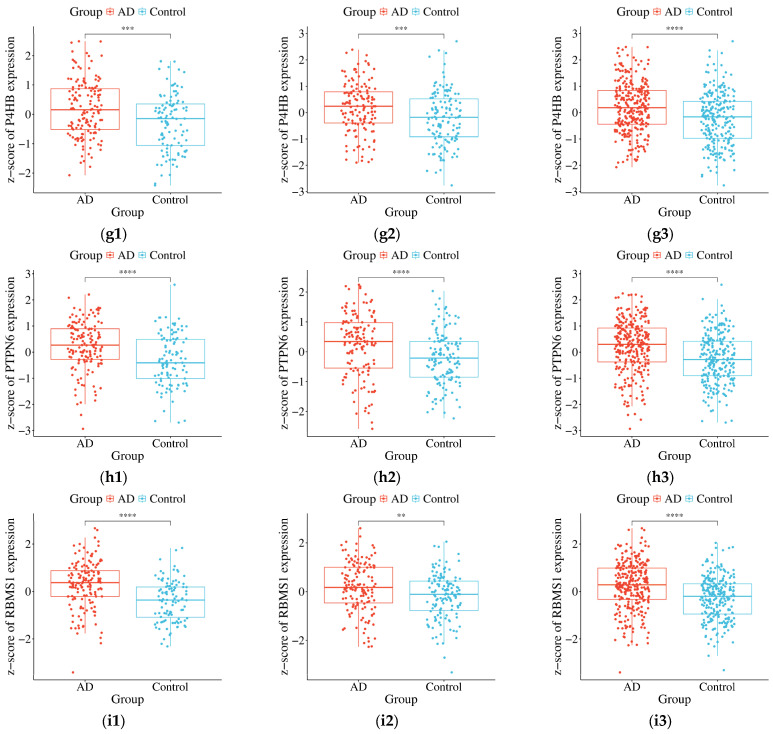
Verification of the FRDEGs expression levels in AD group and control group (**: *p* < 0.01; ***: *p* < 0.001; ****: *p* < 0.0001). (**a1**–**a3**) ACVR1B; (**b1**–**b3**) BRPF1; (**c1**–**c3**) G6PD; (**d1**–**d3**) KLHDC3; (**e1**–**e3**) LAMP2; (**f1**–**f3**) MTCH1; (**g1**–**g3**) P4HB; (**h1**–**h3**) PTPN6; (**i1**–**i3**) RBMS1. Expression data are from the integrated analysis of human peripheral blood samples.

To validate the differential expression of the nine core FRDEGs, this study performed the Wilcoxon test separately within each independent validation dataset (GSE63060 and GSE63061), as well as in the combined validation cohort. The results are presented in Figure 3. The expression levels of each FRDEG (ACVR1B, BRPF1, G6PD, KLHDC3, LAMP2, MTCH1, P4HB, PTPN6, and RBMS1) in the AD group versus the control group are shown for GSE63060 (Figure 3(a1–i1)), GSE63061 (Figure 3(a2–i2)), and the combined cohort (Figure 3(a3–i3)). All nine genes demonstrated statistically significant differential expression (up-regulation) in both independent validation cohorts and in the combined analysis (*p* < 0.05, with specific significance levels indicated as **, ***, or **** in the figure). To quantify the magnitude of these expression differences, the standardized mean difference (SMD, Cohen’s d) and its 95% confidence interval (CI) were calculated for each gene in each dataset. All SMD values were positive (range: 0.48–1.17 in GSE63060; 0.57–0.74 in GSE63061; 0.53–0.88 in the combined cohort), and none of the 95% CIs included zero, confirming a consistent up-regulation pattern with moderate to large effect sizes. This consistent and reproducible up-regulation across two independent datasets robustly confirms the significant association of these FRDEGs with AD status. Their diagnostic potential, based on these expression differences, is further quantified in the following section.

### 3.3. Potential as Candidate Blood-Associated Markers

**Figure 4:**
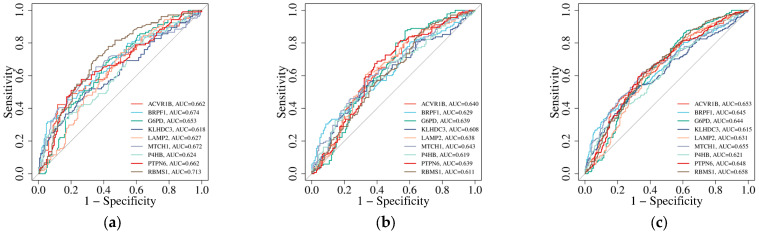
ROC curves evaluating the diagnostic potential of FRDEGs as biomarkers for AD: (**a**) GSE63060 validation cohort; (**b**) GSE63061 validation cohort; (**c**) combined validation cohort. Different line colors represent different FRDEGs. Analysis is based on integrated human blood transcriptome data.

To evaluate the potential of the nine FRDEGs (ACVR1B, BRPF1, G6PD, KLHDC3, LAMP2, MTCH1, P4HB, PTPN6, RBMS1) as candidate blood-associated markers, this work included ROC curve analysis in two independent validation cohorts (GSE63060 and GSE63061), as well as in the combined validation cohort. The results demonstrate that all nine FRDEGs exhibit significant discriminatory ability between AD and control samples across all tested datasets, as shown in Figure 4. In the GSE63060 cohort, the AUC values for ACVR1B, BRPF1, G6PD, KLHDC3, LAMP2, MTCH1, P4HB, PTPN6, and RBMS1 were 0.662 (95% CI: 0.604–0.744), 0.674 (95% CI: 0.619–0.757), 0.653 (95% CI: 0.579–0.720), 0.618 (95% CI: 0.543–0.686), 0.627 (95% CI: 0.561–0.704), 0.672 (95% CI: 0.603–0.744), 0.624 (95% CI: 0.564–0.708), 0.662 (95% CI: 0.584–0.725), and 0.713 (95% CI: 0.642–0.777), respectively. Among these, RBMS1 showed the strongest diagnostic performance with an AUC of 0.713. In the GSE63061 cohort, the nine FRDEGs achieved AUCs of 0.640 (95% CI: 0.577–0.709), 0.629 (95% CI: 0.571–0.703), 0.639 (95% CI: 0.565–0.699), 0.608 (95% CI: 0.549–0.683), 0.638 (95% CI: 0.582–0.714), 0.643 (95% CI: 0.574–0.706), 0.619 (95% CI: 0.558–0.692), 0.639 (95% CI: 0.566–0.698), and 0.611 (95% CI: 0.549–0.683). ACVR1B yielded the highest AUC in this cohort (0.640). To enhance statistical robustness, we further analyzed the combined dataset. The resulting AUCs were 0.653 (95% CI: 0.603–0.714), 0.645 (95% CI: 0.601–0.711), 0.644 (95% CI: 0.593–0.704), 0.615 (95% CI: 0.555–0.667), 0.631 (95% CI: 0.584–0.697), 0.655 (95% CI: 0.605–0.715), 0.621 (95% CI: 0.569–0.682), 0.648 (95% CI: 0.602–0.713), and 0.658 (95% CI: 0.603–0.714). Notably, RBMS1 again demonstrated the highest diagnostic accuracy with an AUC of 0.658. Importantly, the 95% CI for all genes in each cohort did not include 0.5, confirming that their diagnostic performance is statistically significant. These consistent results across independent and combined validation cohorts underscore the reliability and translational potential of these FRDEGs as candidate biomarkers for AD.

### 3.4. High Correlation of FRDEGs

**Figure 5:**
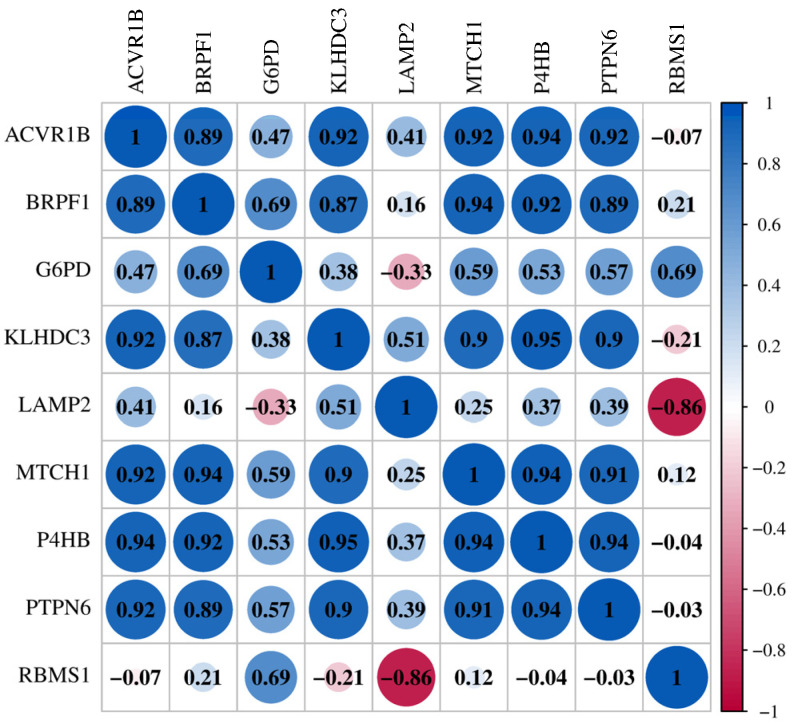
Correlation analysis among FRDEGs (ACVR1B, BRPF1, G6PD, KLHDC3, LAMP2, MTCH1, P4HB, PTPN6, RBMS1). The coefficient of correlation (*r*) is presented by a coloring scheme from red (negative correlation) to blue (positive correlation) while white represents an absence of correlation. The circle increases as the correlation coefficient increases.

The PCC analysis showed there was a strong potential correlation between these nine FRDEGs, as shown in Figure 5. ACVR1B exhibited the strongest positive correlation with P4HB (*r* = 0.94), followed by MTCH1 (*r* = 0.92), PTPN6 (*r* = 0.92), and BRPF1 (*r* = 0.89). Similarly, BRPF1 showed particularly high correlations with KLHDC3 (*r* = 0.87), MTCH1 (*r* = 0.94), P4HB (*r* = 0.92), and PTPN6 (*r* = 0.89). KLHDC3 correlated strongly with MTCH1 (*r* = 0.9), P4HB (*r* = 0.95), and PTPN6 (*r* = 0.9). LAMP2 uniquely exhibited a strong negative correlation with RBMS1 (*r* = −0.86). The correlation between MTCH1 and P4HB was positively correlated (*r* = 0.94), followed by the correlation with PTPN6, which was also positively correlated (*r* = 0.91). Among them, the correlation between P4HB and PTPN6 was the highest (*r* = 0.94). However, the only exception was G6PD, which showed no significant correlations with other FRDEGs. Regardless, the correlation analysis reveals potential high interconnectivity among these key FRDEGs.

### 3.5. Enrichment Analysis of FRDEGs

**Figure 6:**
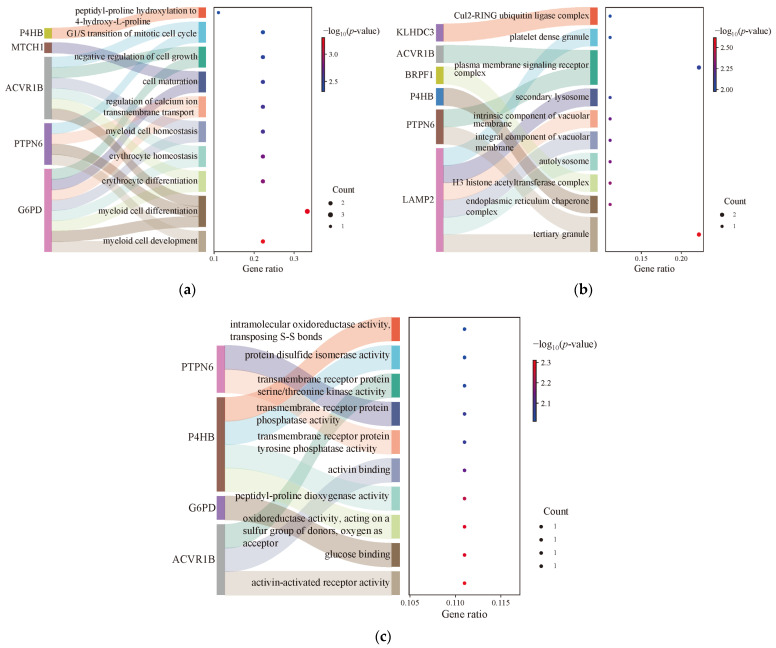
The functional enrichment analysis of FRDEGs: (**a**) biological process; (**b**) cellular component; (**c**) molecular function.

The function analysis of FRDEGs was further investigated, and the results are shown in Table 3 and Figure 6a–c. The biological process mainly contains myeloid cell development, differentiation, and homeostasis, erythrocyte differentiation and homeostasis, and regulation of calcium ion transmembrane transport. Cellular component mainly contains tertiary granule, endoplasmic reticulum chaperone complex, H3 histone acetyltransferase complex, autolysosome, integral/intrinsic component of vacuolar membrane, secondary lysosome, plasma membrane signaling receptor complex, platelet dense granule and Cul2-RING ubiquitin ligase complex. Molecular function mainly contains activin-activated receptor activity, glucose binding, peptidyl–proline dioxygenase activity, and activin binding.

**Figure 7:**
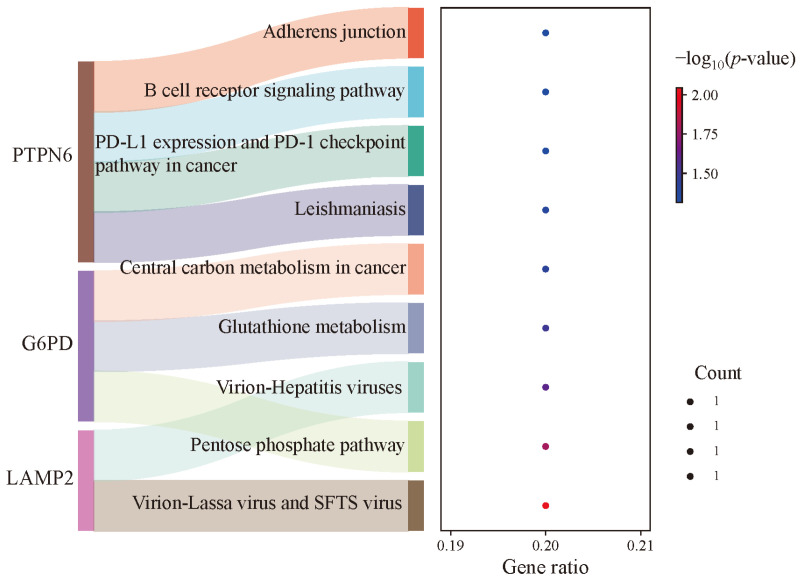
Pathway analysis of FRDEGs.

Pathway analysis identified the involvement of multiple signaling pathways, as summarized in Table 4 and Figure 7. These included metabolic pathways such as the pentose phosphate pathway, glutathione metabolism, and central carbon metabolism. Pathways associated with viral infection included the virion—lassa virus and SFTS virus, and virion—hepatitis viruses. Those related to microbial infection included leishmaniasis. Pathways implicated in immune and inflammatory responses included the PD-L1 expression and the PD-1 checkpoint pathway, and the B cell receptor signaling pathway. Additionally, the adherens junction pathway was also significantly enriched.

### 3.6. FRDEGs-miRNA Interaction Network

**Figure 8:**
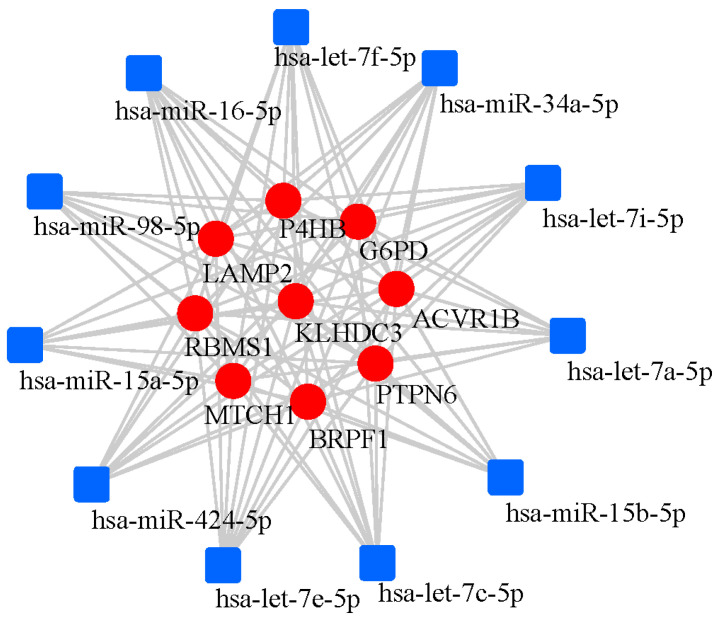
Interactions among 9 hub FRDEGs (ACVR1B, G6PD, KLHDC3, LAMP2, MTCH1, P4HB, PTPN6, RBMS1) and miRNAs. Blue squares represent miRNAs, and red circles represent FRDEGs.

Table 5 summarizes the characteristics and potential roles the nine core FRDEGs. Finally, the FRDEGs and miRNA interaction network was constructed by network analysis. The FRDEGs-miRNA network of ACVR1B, BRPF1, G6PD, KLHDC3, LAMP2, MTCH1, P4HB, PTPN6, and RBMS1 was constructed. The constructed network comprised 528 nodes (miRNAs linked to FRDEGs) and 1457 edges. The specific information is listed in Table S1 of the Supplementary Material. There were 11 miRNAs associated with these 9 hub FRDEGs (ACVR1B, BRPF1, G6PD, KLHDC3, LAMP2, MTCH1, P4HB, PTPN6, RBMS1), namely hsa-let-7a-5p, hsa-let-7c-5p, hsa-let-7f-5p, hsa-let-7e-5p, hsa-let-7i-5p, hsa-miR-15a-5p, hsa-miR-15b-5p, hsa-miR-16-5p, hsa-miR-424-5p, hsa-miR-98-5p, hsa-miR-34a-5p, respectively, as shown in Figure 8.

## 4. Discussion

In order to search for prospective candidate ferroptosis-related genes for AD, this work obtained gene expression profile data and ferroptosis-related genes. Subsequently, nine FRDEGs were identified by integrating ferroptosis-related genes, and the high correlation among these genes was further verified. The significant expression of the core FRDEGs was confirmed, and their potential value in AD development was further identified. Finally, the nine identified FRDEGs (ACVR1B, BRPF1, G6PD, KLHDC3, LAMP2, MTCH1, P4HB, PTPN6, RBMS1) showed significant expression changes, suggesting their potential as ferroptosis-related AD biomarkers. Clinical and histopathological studies have found elevated levels of G6PD in autopsy samples from AD patients [25]. A study reported significantly increased serum G6PD enzymatic activity in AD patients compared to controls [26], suggesting its potential as an AD biomarker. Moreover, LAMP2 and P4HB are associated with autophagy. LAMP2 (lysosomal-associated membrane protein 2) is a highly glycosylated lysosomal membrane protein involved in chaperone protein-mediated autophagy. Approximately 50% of lysosomal membrane proteins belong to the LAMP family, with LAMP2 functioning as a critical regulator of autophagosome–lysosome fusion efficiency [27]. P4HB (prolyl 4-hydroxylase subunit beta) has the functions of oxidation–reduction enzyme, chaperone enzyme and isomerase. It is present in the endoplasmic reticulum, mitochondria, and cytosol, and is an autophagy-related biomarker [28]. It reveals the closed connection among AD, autophagy and ferroptosis. PTPN6 (protein tyrosine phosphatase, non-receptor type 6) is a crucial regulatory protein in the cell signaling process, and is associated with inflammatory responses and cell death. In both innate and adaptive immune systems, PTPN6 significantly modulates cell proliferation and signal transduction [29]. In AD-related studies, it was found that CD33, as a risk factor for AD, can induce PTPN6 activation, thereby regulating the production of pro-inflammatory signals [30]. These points highlight the identification and validation of ferroptosis-related genes as promising AD biomarkers, particularly focusing on G6PD, LAMP2, P4HB, and PTPN6, and their relevance to autophagy and inflammatory pathways in AD. For other genes (ACVR1B, BRPF1, etc.), there are currently few studies on AD. The results of this study suggest that they may be new potential associated factors, and their specific mechanisms of action need further exploration.

Subsequently, the biological functions and pathways of FRDEGs were analyzed. Pathway analysis identified AD-related pathways, including glutathione (GSH) metabolism, pentose phosphate pathway (PPP), adherens junction, and parasite infection-associated pathway (Leishmaniasis). As a major endogenous enzyme-catalyzed antioxidant, GSH has important functions in AD pathogenesis. Although multiple studies suggest decreased GSH levels in the brains of AD patients [31,32], clinical intervention trials aimed at elevating GSH have not demonstrated clear efficacy. This discrepancy may arise from several factors: the decline in GSH might be a late-stage event, where the window for early intervention has already closed; peripherally supplemented GSH precursors may not efficiently cross the blood–brain barrier (BBB); and antioxidant supplementation alone may be insufficient to reverse the established, complex pathological network. This suggests that targeting key regulators of GSH metabolism for intervention may represent a more strategic approach than directly supplementing GSH itself. PPP is a cytoplasmic metabolic process, which is metabolized through the catabolism of glucose-6-phosphate dehydrogenase (G6PD) [33]. In AD, pathologic conditions can alter PPP activity. For example, in brain cells, Aβ protein has been shown to increase the production of ROS, and up-regulates the PPP activity [34]. Furthermore, high levels of ROS can increase autophagy by inducing the inactivation of G6PD and PPP [35]. Autophagy is the process by which cells clear misfolded or damaged macromolecules and organelles, which helps maintain tissue and cell homeostasis. It further modulates extracellular signaling and metabolic stress responses, which is closely related to the pathology of AD [36]. These results suggest that increased ROS and Aβ in AD affect PPP, and high ROS levels also trigger autophagy inactivation, which helps maintain tissue and cell homeostasis.

Another important pathway is the adherens junction, which sustains morphological and functional integrity of the high-dynamic blood–brain and enteric vascular barriers in the human brain. BBB disruption is a hallmark of AD pathogenesis, with adhesion junctions implicated in its dysfunction. One study found that the circulating exosomes in patients’ bodies can mediate the damage of adhesion and connection of BBB receptor vascular endothelial cells, suggesting a new mechanism of peripheral aging exosomes and the risk of AD [37]. So, the disruption of the BBB is implicated in AD progression, and adhesion junction dysfunction might contribute to this. The circulating exosomes related with the BBB are also potential biomarkers for AD. A Leishmania parasite infection pathway (Leishmaniasis) was also identified in this work. Leishmania infection promotes an anti-inflammatory state, including down-regulation of NLRP3 inflammasome activation. Neuroinflammation contributes significantly to AD progression; Leishmaniasis may attenuate neuroinflammatory responses, potentially delaying AD pathogenesis [38,39]. Therefore, neuroinflammation is important in AD progression, and infection might influence its development. These insights highlight that ferroptosis is not an isolated process and may be closely related to multiple pathological links in AD, such as the antioxidant systems, metabolic pathways, autophagy, BBB integrity, exosomes, and the inflammatory-related pathways mentioned above. Furthermore, it should be noted that ferroptosis is a shared pathological process across multiple neurodegenerative diseases, such as Parkinson’s disease and dementia with Lewy bodies. Individual FRDEGs identified in this study may exhibit expression alterations in various disorders. However, the highly interconnected expression correlations among these nine genes and their enrichment in specific pathways (such as the PPP pathway under amyloid stress related to AD, the BBB-related adherens junction pathway) suggest that they may constitute a ferroptosis-related molecular network specific to the AD context. Future studies are needed to validate the diagnostic specificity of this gene set for AD using independent datasets encompassing patients with other neurodegenerative diseases.

**Figure 9:**
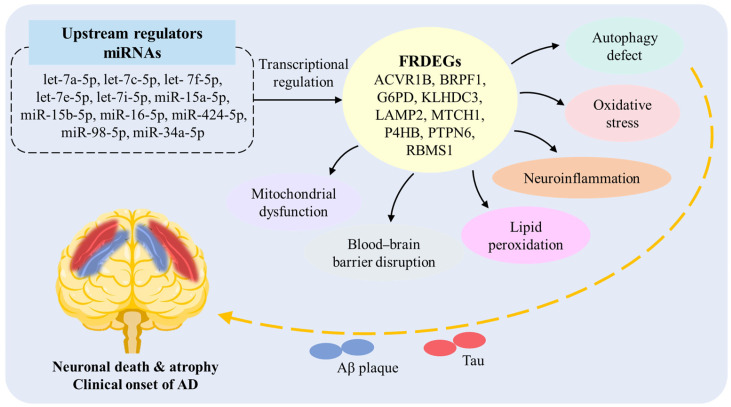
Potential multi-pathway mechanisms of FRDEGs regulatory network involvement in AD pathogenesis.

Finally, this study predicted 11 miRNAs associated with these nine hub FRDEGs (ACVR1B, BRPF1, G6PD, KLHDC3, LAMP2, MTCH1, P4HB, PTPN6, RBMS1) through network analysis. Among them, there are miRNAs from the let-7 family (let-7a/c/f/e/i-5p), and other miRNAs (miR-15a-5p, miR-15b-5p, miR-34a-5p, miR-98-5p, miR-16-5p, miR-424-5p). Among these 11 predicted regulatory miRNAs, such as let-7a/c/f/e and miR-15b/16/424/98/34a-5p, studies have reported their abnormal expression in cerebrospinal fluid (CSF), blood or exosomes of AD patients, and they are associated with pathological features such as Aβ metabolism [40,41,42,43,44,45,46,47,48,49]. AD patients exhibit characteristic let-7 miRNA expression signatures in the CSF. Compared to healthy individuals, let-7b and let-7e levels are significantly higher in the CSF of AD patients [40]. Another study also observed increased let-7f in the CSF of AD patients [41]. Furthermore, serum let-7d-5p and let-7g-5p levels were significantly elevated in AD patients versus controls [42]. In an AD cell model, overexpression of let-7a increased Aβ1-40-induced neurotoxicity by regulating autophagy [43]. Animal experiments revealed that let-7c expression was significantly up-regulated in AD model mice versus controls, leading to the up-regulation of BACE2, which in turn reduced Aβ production [44]. These results highlight a pivotal role for let-7 miRNAs in AD. Moreover, other studies have shown that elevated plasma levels of miR-15b-5p and miR-34a-5p robustly differentiate AD patients [45]. Integrative analyses of miRNA and mRNA datasets in AD blood samples identified key nodes in miRNA-mRNA networks, including miR-34a, miR-15b-5p, and miR-98-5p [46]. Additionally, down-regulation of miR-98-5p has been shown to alleviate Aβ-induced inhibition of cell viability, and suppresses apoptosis in neuronal cells via SNX6 up-regulation [47]. Another study found that miR-16-5p was differentially expressed in the CSF exosomes of young patients with AD [48]. The elevated expression level of miR-424-5p in serum exosomes could distinguish patients with sporadic AD from healthy individuals [49]. However, for some of them such as let-7i-5p and miR-15a-5p, there are currently few direct studies on their roles in AD. Through systematic analysis, this study identified and verified the FRDEGs closely related to AD, and constructed their miRNA regulatory network. Based on the research results of this work and literature review, this study provides a potential multi-pathway mechanism by which the regulatory network of FRDEGs is involved in the pathogenesis of AD, as shown in Figure 9. This suggests that miRNAs may participate in the ferroptosis process of AD by regulating the core target genes, providing new potential candidate molecules for the miRNA regulatory network of AD, which is worthy of further experimental verification.

## 5. Study Limitations and Future Directions

This study also has the following limitations, which point the way for future research. (1) Sample source and tissue specificity: The analysis in this study is based on peripheral blood transcriptomic data. Although peripheral blood is an easily accessible and clinically promising biospecimen, its gene expression profile does not fully mirror that of the core disease site (brain tissue). Expression changes in blood may reflect systemic inflammation, metabolic status, or peripheral immune responses rather than direct neuronal pathology. Future work needs to validate the expression and protein levels of these nine FRDEGs in post-mortem brain tissue and CSF samples from AD patients and controls. This will help confirm whether these markers are directly related to the pathology of the central nervous system and assess their potential as central-specific biomarkers. (2) Cross-sectional design and disease dynamics: This study employs cross-sectional data, which cannot determine whether the expression changes in these genes are early drivers of AD onset or consequences of widespread neuronal death in late-stage disease. This limits our assessment of their value as early diagnostic markers. There is an urgent need to validate these markers in longitudinal cohort studies in the future. For example, in prospective cohorts encompassing cognitively normal individuals, patients with mild cognitive impairment (MCI) and in AD patients, dynamically monitoring the expression of these genes to analyze whether their changes predict conversion from MCI to AD or correlate with the rate of cognitive decline. (3) Associations versus causal mechanisms: This study primarily establishes a statistical association between these FRDEGs and AD status. Although enrichment analysis and literature mining suggest potential biological pathways, bioinformatics analysis itself cannot provide direct causal evidence or elucidate precise molecular mechanisms. Future directions require in-depth functional validation experiments, for instance, in AD cell models, knocking down or overexpressing core genes to examine their effects on ferroptosis sensitivity, Aβ production, tau phosphorylation, and cell viability. (4) Disease specificity and confounding factors: Ferroptosis is a common feature of multiple neurodegenerative diseases and the aging process. This study did not validate the specificity of this set of FRDEGs in other neurodegenerative diseases or healthy aging cohorts. Therefore, it remains unclear whether this gene signature is specific to AD or represents a common response to neurodegeneration. Future research should evaluate the diagnostic specificity of the multi-gene combination constituted by these genes in an independent dataset containing various dementia types and disease controls. Simultaneously, key confounding factors such as age, sex, and comorbidities should be more strictly controlled in the analysis. (5) Lack of association with key clinical–genetic features: Limited by the completeness of information in the public datasets used, this study could not explore the associations between the core FRDEGs and the important genetic risk factor for AD, such as the APOE ε4 allele status, or the severity of clinical symptoms. This information is crucial for assessing the clinical utility and pathological staging value of the markers. Future studies should prioritize cohorts with comprehensive clinical–genetic–imaging multi-modal data. A systematic analysis of the associations between FRDEGs and APOE genotype, brain atrophy patterns, amyloid deposition, and cognitive scale scores is needed to establish their integrated clinical value.

In summary, this study provides new clues and candidate targets for understanding ferroptosis-related molecular events in AD. However, translating these findings into reliable biomarkers or therapeutic targets still depends on subsequent comprehensive research encompassing multi-omics approaches, cross-disease comparisons, longitudinal designs, and rigorous experimental validation.

## 6. Conclusions

This study identified nine FRDEGs, namely, ACVR1B, BRPF1, G6PD, KLHDC3, LAMP2, MTCH1, P4HB, PTPN6, and RBMS1, that are strongly associated with AD and may serve as blood biomarkers related to ferroptosis in AD. Furthermore, this work discovered that multiple miRNAs likely regulate this core ferroptosis-related biomarker network, contributing to the early detection and treatment of AD. These findings hold significant promise for advancing diagnostic and treatment strategies for AD.

## Figures and Tables

**Table 1:** Information on the patient and subject cohorts in the datasets.

Datasets			Patients (*n*)	Subjects (*n*)	All (*n*)
GSE140831	Gender (n)	Male	172	235	407
		Female	166	295	461
		All	338	530	868
	Average age (years)		73	71	71
GSE63060	Gender (n)	Male	46	42	88
		Female	99	62	161
		All	145	104	249
	Average age (years)		75	72	74
GSE63061	Gender (n)	Male	85	54	109
		Female	55	81	166
		All	140	135	275
	Average age (years)		78	75	77

**Table 2:** The complete lists of 9 FRDEGs.

FRDEG	Description	GSE140831		GSE63060		GSE63061	
		FDR	*p*-Value	FDR	*p*-Value	FDR	*p*-Value
ACVR1B	Activin a receptor type 1B	1.07 × 10^−57^	2.39 × 10^−58^	2.30 × 10^−4^	7.43 × 10^−6^	2.13 × 10^−3^	5.59 × 10^−5^
BRPF1	Bromodomain and PHD finger containing 1	9.30 × 10^−64^	3.35 × 10^−65^	4.87 × 10^−5^	1.20 × 10^−6^	1.73 × 10^−3^	4.21 × 10^−5^
G6PD	Glucose-6-phosphate dehydrogenase	2.16 × 10^−61^	2.15 × 10^−62^	6.34 × 10^−3^	3.86 × 10^−4^	4.73 × 10^−3^	1.67 × 10^−4^
KLHDC3	Kelch domain containing 3	1.17 × 10^−58^	2.26 × 10^−59^	6.60 × 10^−3^	4.06 × 10^−4^	4.43 × 10^−2^	3.83 × 10^−3^
LAMP2	Lysosomal associated membrane protein 2	7.58 × 10^−60^	1.17 × 10^−60^	1.13 × 10^−2^	7.92 × 10^−4^	1.01 × 10^−2^	4.76 × 10^−4^
MTCH1	Mitochondrial carrier 1	9.25 × 10^−58^	2.04 × 10^−58^	3.23 × 10^−4^	1.10 × 10^−5^	8.30 × 10^−4^	1.54 × 10^−5^
P4HB	Prolyl 4-hydroxylase subunit beta	6.76 × 10^−60^	1.03 × 10^−60^	4.09 × 10^−3^	2.25 × 10^−4^	1.21 × 10^−2^	6.17 × 10^−4^
PTPN6	Protein tyrosine phosphatase, non-receptor type 6	6.06 × 10^−54^	1.98 × 10^−54^	6.30 × 10^−4^	2.46 × 10^−5^	5.97 × 10^−3^	2.28 × 10^−4^
RBMS1	RNA binding motif single stranded interacting protein 1	5.89 × 10^−64^	1.90 × 10^−65^	2.56 × 10^−6^	4.00 × 10^−8^	1.80 × 10^−2^	1.07 × 10^−3^

**Table 3:** Function analysis.

GO Analysis	ID	Description	*p*-Value	Gene
Biological process	GO:0061515	myeloid cell development	0.0005	G6PD, PTPN6
	GO:0030099	myeloid cell differentiation	0.0006	ACVR1B, G6PD, PTPN6
	GO:0030218	erythrocyte differentiation	0.0014	ACVR1B, G6PD
	GO:0034101	erythrocyte homeostasis	0.0016	ACVR1B, G6PD
	GO:0002262	myeloid cell homeostasis	0.0024	ACVR1B, G6PD
	GO:1903169	regulation of calcium ion transmembrane transport	0.0025	G6PD, PTPN6
	GO:0048469	cell maturation	0.0029	G6PD, MTCH1
	GO:0030308	negative regulation of cell growth	0.0034	ACVR1B, G6PD
	GO:0000082	G1/S transition of mitotic cell cycle	0.0044	ACVR1B, PTPN6
	GO:0018401	peptidyl–proline hydroxylation to 4-hydroxy-L-proline	0.0048	P4HB
Cellular component	GO:0070820	tertiary granule	0.0024	LAMP2, PTPN6
	GO:0034663	endoplasmic reticulum chaperone complex	0.0046	P4HB
	GO:0070775	H3 histone acetyltransferase complex	0.0046	BRPF1
	GO:0044754	autolysosome	0.0051	LAMP2
	GO:0031166	integral component of vacuolar membrane	0.0055	LAMP2
	GO:0031310	intrinsic component of vacuolar membrane	0.0055	LAMP2
	GO:0005767	secondary lysosome	0.0078	LAMP2
	GO:0098802	plasma membrane signaling receptor complex	0.0082	ACVR1B, PTPN6
	GO:0042827	platelet dense granule	0.0096	LAMP2
	GO:0031462	Cul2-RING ubiquitin ligase complex	0.0105	KLHDC3
Molecular function	GO:0017002	activin-activated receptor activity	0.0049	ACVR1B
	GO:0005536	glucose binding	0.0054	G6PD
	GO:0016670	oxidoreductase activity, acting on a sulfur group of donors, oxygen as acceptor	0.0054	P4HB
	GO:0031543	peptidyl–proline dioxygenase activity	0.0059	P4HB
	GO:0048185	activin binding	0.0073	ACVR1B
	GO:0005001	transmembrane receptor protein tyrosine phosphatase activity	0.0083	PTPN6
	GO:0019198	transmembrane receptor protein phosphatase activity	0.0083	PTPN6
	GO:0004675	transmembrane receptor protein serine/threonine kinase activity	0.0093	ACVR1B
	GO:0003756	protein disulfide isomerase activity	0.0098	P4HB
	GO:0016864	intramolecular oxidoreductase activity, transposing S-S bonds	0.0098	P4HB

**Table 4:** Pathway enrichment analysis.

ID	Description	*p*-Value	Gene
hsa03273	Virion—Lassa virus and SFTS virus	0.0090	LAMP2
hsa00030	Pentose phosphate pathway	0.0164	G6PD
hsa03272	Virion—Hepatitis viruses	0.0253	LAMP2
hsa00480	Glutathione metabolism	0.0310	G6PD
hsa05230	Central carbon metabolism in cancer	0.0372	G6PD
hsa05140	Leishmaniasis	0.0413	PTPN6
hsa05235	PD-L1 expression and PD-1 checkpoint pathway in cancer	0.0470	PTPN6
hsa04662	B cell receptor signaling pathway	0.0475	PTPN6
hsa04520	Adherens junction	0.0485	PTPN6

**Table 5:** Characteristics and potential roles of the nine core FRDEGs in AD.

FRDEG	Description	Expression in AD	Diagnostic Potential (AUC)	Potential Role/Pathway in AD
ACVR1B	Activin a receptor type 1B	Up-regulated	0.662, 0.640, 0.653	myeloid cell/erythrocyte differentiation, erythrocyte/myeloid cell homeostasis, negative regulation of cell growth, G1/S transition of mitotic cell cycle, plasma membrane signaling receptor complex, activin binding, transmembrane receptor protein serine/threonine kinase activity
BRPF1	Bromodomain and PHD finger containing 1	Up-regulated	0.674, 0.629, 0.645	H3 histone acetyltransferase complex
G6PD	Glucose-6-phosphate dehydrogenase	Up-regulated	0.653, 0.639, 0.644	myeloid cell development, myeloid cell/erythrocyte differentiation, erythrocyte/myeloid cell homeostasis, regulation of calcium ion transmembrane transport, cell maturation, negative regulation of cell growth, glucose binding
KLHDC3	Kelch domain containing 3	Up-regulated	0.618, 0.608, 0.615	Cul2-RING ubiquitin ligase complex
LAMP2	Lysosomal associated membrane protein 2	Up-regulated	0.627, 0.638, 0.631	tertiary granule, autolysosome, integral/intrinsic component of vacuolar membrane, secondary lysosome, platelet dense granule
MTCH1	Mitochondrial carrier 1	Up-regulated	0.672, 0.643, 0.655	cell maturation
P4HB	Prolyl 4-hydroxylase subunit beta	Up-regulated	0.624, 0.619, 0.621	peptidyl-proline hydroxylation to 4-hydroxy-L-proline, endoplasmic reticulum chaperone complex, oxidoreductase activity, acting on a sulfur group of donors, oxygen as acceptor, peptidyl-proline dioxygenase activity, protein disulfide isomerase activity, intramolecular oxidoreductase activity, transposing S-S bonds
PTPN6	Protein tyrosine phosphatase, non-receptor type 6	Up-regulated	0.662, 0.639, 0.648	myeloid cell development and differentiation, regulation of calcium ion transmembrane transport, G1/S transition of mitotic cell cycle, tertiary granule, plasma membrane signaling receptor complex, transmembrane receptor protein tyrosine phosphatase activity
RBMS1	RNA binding motif single stranded interacting protein 1	Up-regulated	0.713, 0.611, 0.658	RNA metabolism and post-transcriptional regulation

## Data Availability

The GSE140831, GSE63060 and GSE63061 gene expression profiles were downloaded from the GEO database (http://www.ncbi.nlm.nih.gov/geo/ (accessed on 5 February 2026)). Ferroptosis-related genes were derived from the FerrDB (http://www.zhounan.org/ferrdb/current/ (accessed on 5 February 2026)).

## Supplementary Materials

The following supporting information can be downloaded at: https://www.mdpi.com/article/10.3390/genes17020224/s1, Table S1: The miRNAs related to FRDEGs (A total of 483 nodes and 1251 edges).
